# Textile Organic Electrochemical Transistors as a Platform for Wearable Biosensors

**DOI:** 10.1038/srep33637

**Published:** 2016-09-26

**Authors:** I. Gualandi, M. Marzocchi, A. Achilli, D. Cavedale, A. Bonfiglio, B. Fraboni

**Affiliations:** 1 Dipartimento di Fisica e Astronomia, Università di Bologna, Viale Berti Pichat 6/2, 40127 Bologna, Italy; 2 Dipartimento di Ingegneria Elettrica ed Elettronica, Università di Cagliari, Piazza D’Armi, 09123 Cagliari, Italy.

## Abstract

The development of wearable chemical sensors is receiving a great deal of attention in view of non-invasive and continuous monitoring of physiological parameters in healthcare applications. This paper describes the development of a fully textile, wearable chemical sensor based on an organic electrochemical transistor (OECT) entirely made of conductive polymer (PEDOT:PSS). The active polymer patterns are deposited into the fabric by screen printing processes, thus allowing the device to actually “disappear” into it. We demonstrate the reliability of the proposed textile OECTs as a platform for developing chemical sensors capable to detect in real-time various redox active molecules (adrenaline, dopamine and ascorbic acid), by assessing their performance in two different experimental contexts: i) ideal operation conditions (i.e. totally dipped in an electrolyte solution); ii) real-life operation conditions (i.e. by sequentially adding few drops of electrolyte solution onto only one side of the textile sensor). The OECTs response has also been measured in artificial sweat, assessing how these sensors can be reliably used for the detection of biomarkers in body fluids. Finally, the very low operating potentials (<1 V) and absorbed power (~10^−4 ^W) make the here described textile OECTs very appealing for portable and wearable applications.

Wearable technology is the branch of technology developing devices that can be worn and, at the same time, that contain advanced electronic circuits, not only for incorporating practical functions, but also for purely aesthetic reasons. Wearable technology is now applied to the production of different special garments such as protective clothing, soldier uniforms, and activity trackers^1^. Furthermore, there is a tremendous and growing interest in developing wearable technology for physiological monitoring in order to obtain a novel class of personalized point-of-care devices that could be extensively and effortlessly integrated into the daily life of a patient in the form of wireless body sensors^1^,^2^,^3^. A wearable sensor should display suitable mechanical features (flexibility, light weight and stretch ability), washability, low power requirements and should be comfortable when worn^1^. So far most research efforts in this direction have been focused on the production of miniaturized wearable appliances based on relatively mature technologies such as motion tracking^4^,^5^, bio-electrical signals analysis and temperature detection^6^.

Analysis of human biofluids is considered a valuable diagnostic tool for a number of diseases. Blood analysis is considered as the most reliable of these techniques, however it is intrinsically invasive, as blood must be extracted from the body for being analyzed. Other biofluids are considered interesting for performing biomonitoring. Among them, sweat analysis allows evaluating several physical abnormal conditions, both pathological or not, as for instance doping^7^. This is the reason why methods for determining sweat composition by means of portable, or even wearable systems are highly desirable.

Although the continuous monitoring of bio-compounds would pave the way to a dramatically wider field of applications, the number of effective wearable sensing devices reported in the literature is limited^8^. The electrochemical transduction is widely used, but optical and magnetic sensors are also employed^3^. Different wearable sensors are developed to operate in different liquid matrices such as interstitial fluid^9^,^10^, breath^11^,^12^,^13^, sweat^14^,^15^,^16^, saliva^17^ and tears^18^,^19^.

The glucose determination is an important market driver in developing wearable sensors because 35 million diabetes patients need to control glycemia. Glucose sensors are already available on the market, but they are still quite invasive as they require electrode implantation or relatively bulky readout electronics.

To overcome these drawbacks, a promising solution is the monitoring of biomarkers in external biological fluids (e. g. sweat, saliva and urine) through wearable sensors. These devices can be embedded in the textile to become truly non-invasive and less perceptible, as they do not require direct contact with blood, with the further significant advantage of maintaining hygienic conditions (no implantation). Few recent papers proposed some solutions for developing such kind of devices. PEDOT:PSS^20^,^21^,^22^,^23^ and carbonaceous materials^15^,^24^ are the main materials which are used to deposit conductive tracks on a textiles.

Organic electrochemical transistors (OECTs) are devices^25^,^26^ that exhibit features that are very fascinating for the design of wearable sensors. An OECT is composed by a stripe of conductive polymer that works as a channel, and by another electrode that works as a gate (Fig. 1A). Between them is placed an electrolyte solution. The channel current can be modulated by the gate voltage through electrochemical reactions that change the charge-carrier concentration in the transistor channel material and, consequently, the conductivity of the channel (Fig. 1B,C). Since the transistor architecture is the combination of a sensitive element and an amplifier, OECTs directly amplify the electrochemical signal. Moreover, OECTs do not require a three electrode setup, making these device and their readout electronics simpler than the potentiostats that are commonly used as electrochemical sensors. Therefore the OECT structure can be easily embedded in a flexible fabric and a 3D structured substrate, because it does not require a metal reference electrode and a counter electrode that are required by standard electrochemical sensors. Finally, the electrochemical processes take place at potentials lower than 1 V, thus allowing for very low power supply and portable devices. The literature clearly shows the potentiality of OECTs as sensors. In fact, several no-textile OECTs, all endowed with a metal gate electrode, have been developed in order to measure the concentration of different chemical compounds such as cations^27^,^28^, dopamine^29^, adrenaline^30^, ascorbic acid^31^ and glucose^32^,^33^,^34^.

These features make OECTs a winning technology for developing wearable devices. Preliminary examples of textile OECTs have been reported using conductive yarns that, however, do not possess the electrical transport properties needed to be used as sensors^22^,^35^. Iannotta *et al*.^30^,^36^ has proposed a partly-textile Organic electrochemical transistors (OECT) composed by a PEDOT:PSS channel and a metal gate electrode for the detection of ions and adrenaline. However, large improvements are still needed to clearly demonstrate the applicability and the full potential of these devices.

This paper describes the development of a fully textile, wearable OECT sensor for biomarker determination in external biological fluids without the requirement of an invasive electrode implantation. The channel and the gate were both obtained by depositing PEDOT:PSS directly onto the fabric, and thus all conductive tracks of device are made by polymer. Consequently, the sensor is truly embedded into the textile, actually “disappearing” into it. The OECTs we propose are fabricated using screen printing, a technology that is widely employed in the textile industry and can be easily scaled to industrial processes. Therefore our device can be also deposited directly on a garment in order to give it a new smart functionality. We demonstrate the reliability of the proposed textile OECTs as a platform for developing chemical sensors by assessing the detection of various redox active molecules (adrenaline, dopamine and ascorbic acid), also in artificial sweat, as an example of external biological fluid also used as a standard in textile industries.

## Results

Textiles functionalized with PEDOT:PSS can be prepared by dipping the pristine fabric in a suspension of conductive polymer in order to cover the textile surface with particles of conductive polymer. The following drying step removes the solvent, leaving a thin film of polymer on the fibers. An organic solvent, called secondary dopant, is often added to the commercial suspension of PEDOT in order to increase its electrical conductivity. Finally other chemical compounds can be added to the suspension to endow with specific features the conductive polymer layer. An example of such compounds is (3-Glycyloxypropyl)trimethoxysilane (GOPS) that is a cross-linker often added to the PEDOT suspension for increasing its stability in aqueous environment. We used a PEDOT:PSS formulation that is composed by one part of ethylene glycol and two parts of Clevios PH 1000, wherein, after the mixing, 1% of GOPS was added.

The commercial PEDOT:PSS suspension is not suitable for directly carrying out screen printing, because the polymer particles can spread into the fabric by capillarity leading to the formation of active patterns with an ill-defined shape. An appropriate amount of the solvent was evaporated from the PEDOT:PSS suspension before deposition, in order to obtain a viscous suspension that allows to print patterns with high lateral resolution. This PEDOT:PSS ink has the mechanical features of a gel and was used for screen printing the patterns of conductive polymer onto the textile by employing a stencil with a suitable shape and a fill blade.

**Figure 2:**
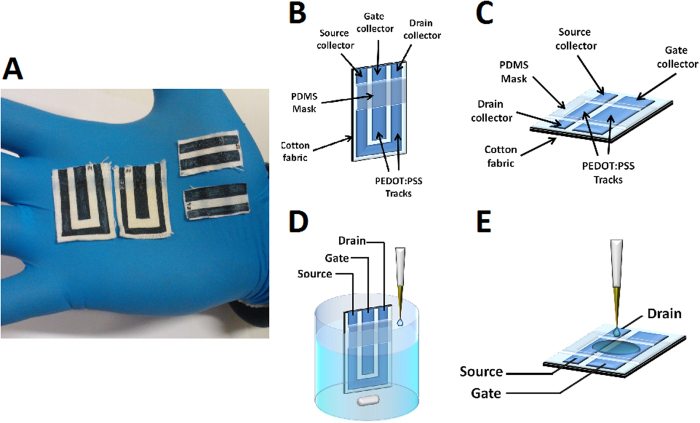
Device pictures and experimental setup. (**A**) Pictures of screen printed OECTs obtained in the conformation G1 (1) and G2 (2). (**B**) Scheme of OECT in G1 geometry. (**C**) Scheme of OECT in G2 geometry. (**D**) Scheme of experimental apparatus for G1 transistor. (**E**) Scheme of experimental apparatus for G2 transistor.

The devices were prepared with two different geometries (G1 and G2) that are reported in Fig. 2. It is worthy to note that the whole OECT structure here presented is made by PEDOT:PSS and thus no metal electrodes are needed that could hinder an optimal conformability to flexible 3D structure of the fabric. The PEDOT:PSS patterns display a well-defined shape that is clearly visible onto the fabrics thanks to the typical blue color of PEDOT:PSS. The sheet resistance of PEDOT:PSS-modified textile resulted equal to 38 ± 7 Ω/□, and this value is much lower than the one of the pristine textile (3.2 ± 0.3 10^10^ Ω/□). This value is also lower than those reported in literature for conductive textiles (Table 1) that have been used for the production of OECTs. Such results indicate that the screen printing of PEDOT:PSS is a promising technique to deposit PEDOT:PSS on a textile and the performance are good enough to produce an OECT. Our conductive fabrics are also comparable with those used to realize amperometric sensors embedded in garments (see Table 1), suggesting that PEDOT:PSS modified textiles can be also used for this application. Such evidence is very important also for the operation of the OECT, because the sensing element of the transistor has a response that is ruled by the same chemical and physical parameters of the amperometric sensing process.

It is worth noting that the electrical performance of the conductive patterns is maintained after and during a mechanical deformation of the devices. The resistance of the OECT channel with geometry G1 was measured while the device was bent around a rod of 7.5 mm of diameter. No significant variation was observed during the process, suggesting that the device maintains its electrical properties despite the deformation (see Supplementary Information Fig. S2).

In order to produce a wearable device, a very significant issue is its washability. This property was evaluated for our OECT printed sensors by submitting them to several hand-washing cycles at T = 35 °C and measuring their sheet resistance after every step. In Fig. S3 the sheet resistance of the textile as a function of the number of washing cycles is reported. The electrical properties of PEDOT-modified textile change after the first washing steps, but the value reached after two cycles (about 70 Ω/□) remains constant after the following washings.

### OECT in G1 layout

We first tested the sensing ability of textile OECTs with a geometry (G1, see Fig. 2B,D) similar to that of chemical sensors we previously realized on plastic and glass substrates^31^. The channel has a U shape that follows the border of the device, whereas the gate is a straight stripe placed between the source and drain electrodes, as shown in Fig. 2. Such a conformation enables the immersion of the transistor in a solution whereas the electrode terminals are kept dry.

**Figure 3:**
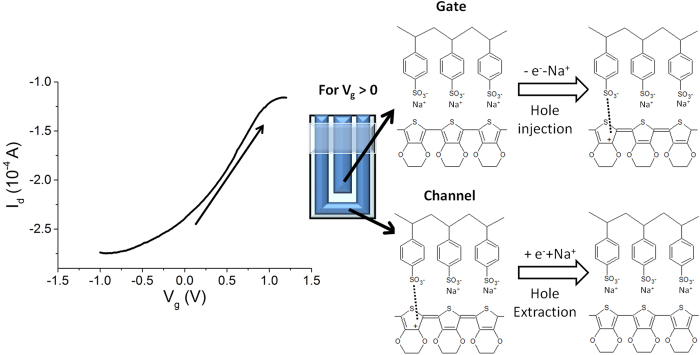
Electrical characterization of device in G1 geometry. I_d_-V_g_ curves (V_d_ = −0.3 V) recorded for a textile OECT transistor in configuration G1, dipped in PBS solution.

The textile OECT was dipped in PBS (0.1 M phosphate buffer saline pH 5.5) and the transfer and characteristic curves were acquired (Fig. 3 and Fig. S4). At the lowest V_g_ value, −1 V, the highest I_d_ (in modulus) was recorded as expected^31^.

As V_g_ increases, the PEDOT:PSS that forms the gate gets oxidized, electrons are extracted from the transistor channel material and, consequently, holes are formed (Fig. 3). In order to keep the electroneutrality of the material, cations move from the gate to the electrolyte solution. At the same time, PEDOT:PSS in the channel is reduced, electrons are injected in the transistor channel material and the recombination between electrons and holes occurs. In such a case the cations move from the electrolyte solution to the channel to ensure electroneutrality. Since holes are the charge carriers in PEDOT:PSS, the increase in V_g_ leads to a decrease in channel conductivity. For high applied V_g_ the channel partly maintains its conductivity, probably because the complex 3D structure of the fabric substrate partially hinders ion diffusion into the conductive polymer and thus holes cannot be completely extracted from PEDOT:PSS layer. Such results clearly show that V_g_ can control the current that flows between the source and the drain electrodes, hence the device operates as a transistor.

Any type of redox process can affect the OECT behavior and, consequently, such phenomena can be used to detect redox active compounds. We investigated the sensing features of our fully textile OECTs in G1 geometry by evaluating its response to ascorbic acid, dopamine and adrenaline. The textile all-PEDOT OECT takes advantage of the electrocatalytic features of the conductive polymer which can oxidize such biocompounds.

**Figure 4:**
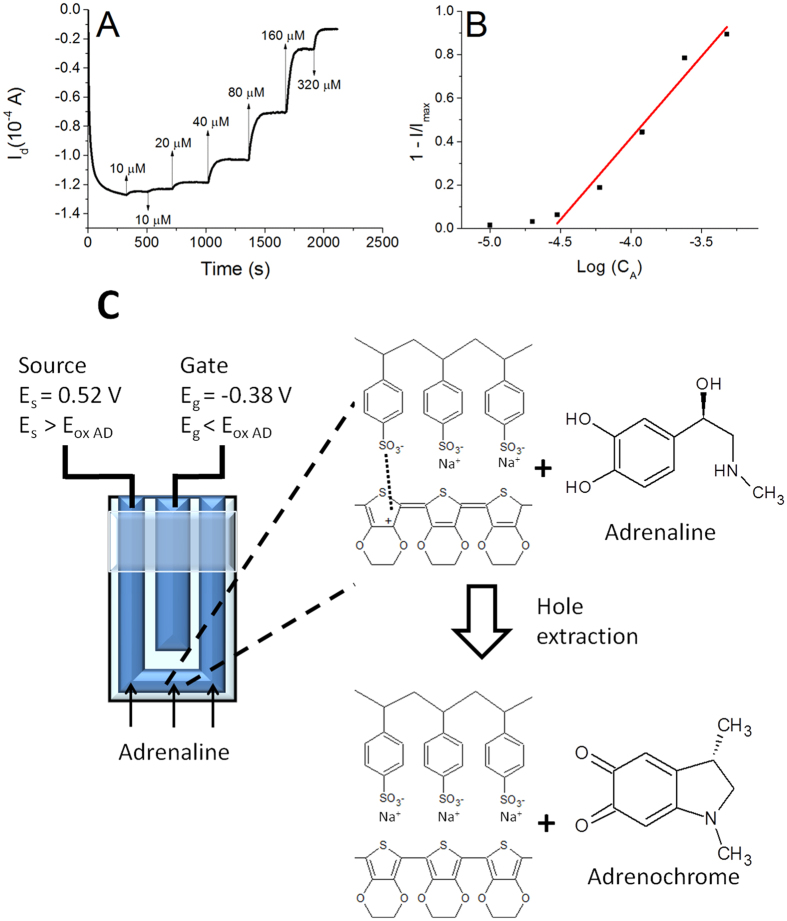
Response of a textile OECT in configuration G1. (**A**) I_d_ vs. time curve (V_g_ = −0.9 V; V_d_ = −0.3 V) obtained after the addition of different adrenaline amounts. The additions are labeled with arrows indicating the increase of concentration. (**B**) 1 − I/I_max_ vs. LogC_AA_ plot. (**C**) Working principle of textile OECTs.: the PEDOT:PSS channel exhibits a potential which is high enough to electro-oxidize adrenaline. The reaction is also reported in simplified form in the panel according to Coppedè *et al*.^20^. The oxidation leads to hole extraction from the transistor channel material and, thus, I_d_ decreases.

The transistors were soaked in PBS solution under stirring (Fig. 2D), and the gate and the drain were biased at −0.9 V and −0.3 V, respectively. These values were optimized in a previous work^31^ for an all-PEDOT:PSS OECT sensor obtained on a glass slide substrate. Since oxidizable compounds are detected by OECTs through a decrease of the drain current, a highly conductive channel ensures a high sensitivity and a wide linear range because of the high starting I_d_ obtained. The chosen gate voltage ensures a quick and stable response. Figure 4A shows the I_d_ *vs* time plot that was recorded from a textile OECT in configuration G1 while different amounts of adrenaline were added in the electrochemical cell. Adrenaline, dopamine and ascorbic can be detected by the sensor because they are oxidizable compounds and, consequently, they react with the positively-biased PEDOT:PSS channel with an electro-catalytic pathway^37^. As a further evidence of the proposed mechanism, the electrochemical potential of the source collector was measured with respect to a Saturated Calomel Electrode before starting the analyte additions and resulted equal to 0.52 V. Consequently, the gate electrochemical potential was −0.38 V. These data point out that only the PEDOT:PSS located in the channel exhibits an electrochemical potential that is high enough (>E_ox_ of analytes) to electro-catalyze the oxidation of the here examined biomolecules^37^ (Fig. SI7 for adrenaline). The reaction between adrenaline and PEDOT is reported (Fig. 4).

The analytes give electrons to PEDOT:PSS in the channel and the consequent recombination between electrons and hole leads to a decrease of the channel conductivity. The variation of hole concentration can be monitored during the sensing process also by following the variation of the electrochemical potential of the source electrode^31^.

This decrease in the drain current has been reported as 1 − I/I_max_ in order to obtain a normalized value that can be compared with other devices. The measured 1 − I/I_max_ values linearly depend on the logarithm of adrenaline concentration in the range 3 10^−5^–5 10^−4 ^M and the line exhibits a slope of 0.75 ± 0.01 decade^−1^ and a R^2^ equal to 0.97. For lower concentrations the trend is not linear, but a response to the adrenaline additions can be observed down to 1 10^−5^ M. Calibration plots with a similar shape have been recently reported in literature^38^ for OECTs fabricated on plastic or glass substrates and with a metal gate electrode, assessing that the here described all-PEDOT textile devices exhibit a behavior comparable to non-textile ones. The response time was evaluated as the time required to reach 90% of the maximum current after each adrenaline addition and resulted equal to 70 s, highlighting that the device can react to the variation of adrenaline concentration almost in real time. Finally, after stabilization, the signal shows no drift, confirming a good stability of the transistor. Since similar responses have been also obtained for ascorbic acid and dopamine, it is clearly demonstrated that the here proposed sensors can work for detecting redox active compounds. The parameters obtained from the calibration plot of all compounds are reported in Table 2 and the graphs are shown in the Supplementary Information.

Summarizing, the all-PEDOT OECTs we fabricated on textile work as regular OECTs with a metal gate electrode when tested in ideal conditions, i.e. when completely immersed in a solution that contains the target compounds (geometry G1, see Fig. 2).

It is noteworthy that the sensitivity of textile OECTs is higher than the one observed for similar non-textile OECTs, fabricated on a glass substrate, as can be also evaluated from the slope of the normalized (1 − I/I_max_) currents (see Table 2). Gualandi *et al*.^31^ recently reported on all-PEDOT OECTs fabricated on glass, and the sensitivity of such devices are also reported in Table 2 for comparison. In all cases the textile OECT exhibits higher sensitivities than the glass one. This behavior can be explained by considering that, when PEDOT:PSS is deposited on a textile, it conformably covers the three-dimensional structure of its fibers, obtaining a large surface-to-volume ratio if compared to devices prepared on a glass substrate. This larger electroactive area of textile PEDOT:PSS leads to a higher electrochemical signal that is then amplified by the transistor architecture.

**Figure 5:**
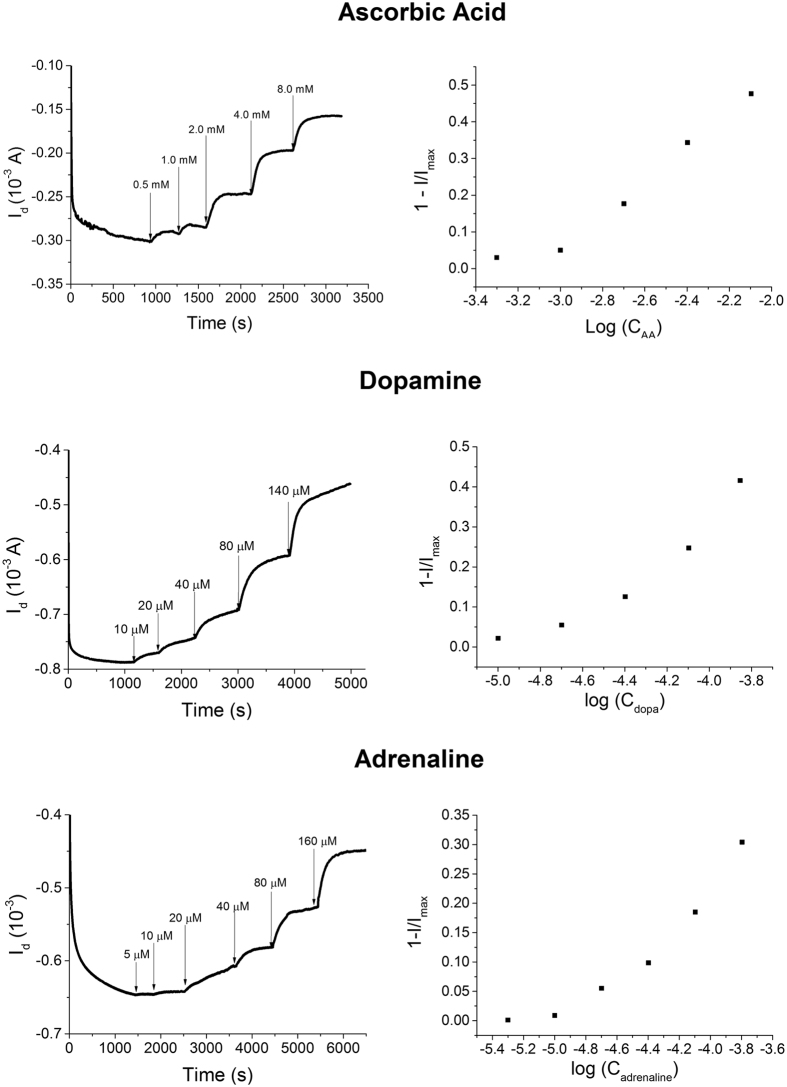
OECT response in artificial sweat. Response of a textile G1 OECT and its calibration plot obtained while different amounts of adrenaline, dopamine and ascorbic acid were added to the artificial sweat.

The sensor response was also studied in artificial sweat (ISO 105-E04-2008E pH 8.0)^39^ in order to verify its performance in a biological fluid. The calibration plots (Fig. 5) exhibit a shape that is very similar to the one obtained in PBS, confirming the potential for the application of our fully textile OECT in monitoring biomarkers contained in body perspiration. Table 1 SI reports the main parameters associated to the calibration plots obtained in sweat.

### OECT in G2 layout

**Figure 6:**
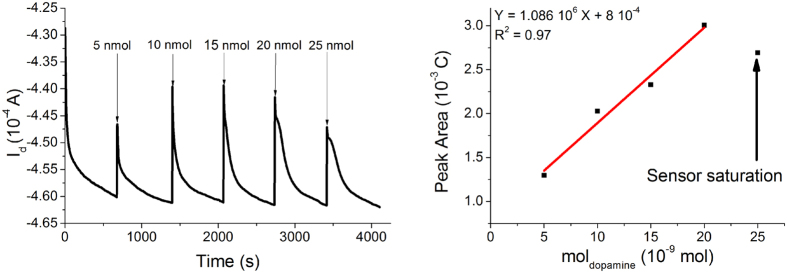
Response of a textile OECT in geometry G2. (**A**) I_d_ vs time curves (V_g_ = −0.9 V; V_d_ = −0.3 V) obtained after the addition of different dopamine amounts. The additions are labeled with arrows where the amount of added dopamine is reported. (**B**) Charge vs. mol_Dopamine_ plot.

Since in wearable applications the sensor cannot be completely dipped in a solution that contains the target compound (for instance when the OECT is integrated in the t-shirt of an athlete), we designed and tested a different device geometry, labeled G2 (Fig. 2C,E) to assess its performance in real-life conditions. The G2 OECT is composed by two parallel rectangular PEDOT:PSS stripes (gate and channel). The electrolyte is confined by a PDMS well in a small area that partly covers both the channel and the gate, in order to minimize the electrolyte volume used in the transistor and to grant an effective and reliable performance in real-life applications. The OECT in the geometry G2 was assessed by adding 10 μL of PBS in the area between the gate electrode and channel to simulate the wetting of fabric due to sweat. The OECT characteristic curves demonstrate (Fig. S8) that the gate potential controls the drain current proving that the little amount of added electrolyte solution is enough to ensure both the electrical contact between the two PEDOT:PSS track and the occurrence of the redox processes required for transistor operation. The sensing ability of OECT in geometry G2 was investigated by adding small amounts of analyte solutions on the operating device. Figure 6 shows the I_d_ vs time graph obtained when dopamine was used as redox active compound. The OECT responds to the additions of redox active molecules with a decrease of I_d_ as previously observed for the ideal conditions (geometry G1). However, after the initial signal variation, I_d_ slowly increases (Fig. 6) until the baseline is again reached: the effect of this behavior is the presence in the I-t curve of several peaks, whose area is proportional to the amount of added analyte. Such a behavior can be explained by supposing that, after the initial current decrease, the analyte is depleted from the electrolyte by the electrochemical reactions which are at the basis of sensor transduction. Such effect cannot be observed in ideal conditions (geometry G1) because in that case the solution close to the transistor surface is continuously renovated. In order to verify this hypothesis, we have calculated the charge that flows at the gate electrode after each dopamine addition. The obtained values are very close to those expected from the dopamine electroxidation process, considering two exchanged electrons for each molecules (see Table S2). Therefore the I_d_ decrease can be explained by an actual change of composition of the electrolyte onto the sensor surface. The peak area linearly depends on the number of analyte moles added on the transistor. The slope of calibration plot is equal to (1.1 ± 0.1) 10^5^ C mol^−1^ with a R^2^ of 0.985 and we can thus use this sensor to extract real-time quantitative data on the analyte concentration in the electrolyte.

Similar responses have been also obtained for ascorbic acid and adrenaline (Table 2) and the experimental response curves are reported in the Supplementary Information (Figs S9 and S10). Summarizing the results we have obtained on the wearable OECT sensors in geometry G2 (i.e. in conditions that are more close to real-life applications, with a very low volume of electrolyte solution only on one side of the fabric) demonstrate that our fully textile devices can detect the variation of redox-active compounds also in this configuration, even if with a slightly lower sensitivity.

Finally, it is worth noting that the here reported fully-textile OECTs exhibit a low adsorbed power (~1.5 10^−4^ W for both G1 and G2 configurations) which is comparable to the values reported in literature for other wearable devices (Table 3). Since in the present work we focus our attention on the sensing element, we report the energy consumption due to the current that flows in the transistor channel (P = V_d_ I_d_). The comparison with other devices shows that amperometric or optical sensors adsorb an electrical power that is higher than for OECTs, while the potentiometric sensor works without energy supply^40^ and, thus, it exhibits no energy consumption. The data reported in Table 3 indicate how the limited power adsorption of fully textile OECT paves the way to its use as a wearable device.

## Conclusion

Chemical sensing of biomarker in body fluids is a promising analytical tool that could be integrated in wearable products, with a consequent large impact on the development of devices able to continuously monitor the health parameters of people and patients. The essential issue that must be addressed for a real application is unobtrusiveness of the garment itself. In order to accomplish this need, we have proposed a new OECT sensor that is completely embedded in a textile to produce a sensor which detects redox active biomolecules in external biological fluid. Moreover our fully textile OECT sensor is based on PEDOT:PSS (active channel and gate electrode), and is processable in one single step via screen printing processes. This evidence is a further important technological advantage because screen printing is a soft technique that ensures a low consumption of conductive ink (ideally the whole printed amount is used for modifying the textile). Furthermore this technique is already widely exploited by textile industry, thus making the industrialization of such device easy and quick.

The here reported sensors were tested using three redox-active biomolecules (ascorbic acid, adrenaline and dopamine). Their performance is very close to that of non-textile OECTs, proving that the OECT technology has been successfully transferred onto a textile substrate that exhibits a complex 3D structure with respect to the flat substrate commonly used to produce OECT sensors. The OECT response to neurotransmitters (adrenaline and dopamine) has also been measured in artificial sweat, thus demonstrating that the sensor can be used for the detection of biomarkers in external body fluids without the electrode implantation.

Starting from the configuration which we usually adopt for non-textile sensors (G1), we developed a new electrode structure (G2) that operates with a very little amount of electrolyte solution (few micro liters) in order to obtain a device that can work by taking advantage of the natural human perspiration. The little electrolyte volume slightly modifies the sensor operation mode, because the biomolecules are consumed by the electrochemical reaction which is at the basis of OECT sensing (Figs 4 and 6). Consequently the OECT exhibits a response with peaks in correspondence of every analyte additions.

Finally, the textile OECTs we here described exhibit other very appealing features for wearable sensors: 1) the operating potentials are very low (<1 V), a key point considering that the device must be placed in direct contact with the skin; 2) since the current used as an output signal is quite high (~1 mA), it requires a simple readout electronics; 3) the absorbed power is very low (~10^−4^ W) and such feature is very suitable for the development of portable applications 4) it can be deformed without observing a degradation of its electrical performance; 5) the OECT stability was assessed under repetitive hand-washing cycles. All these features make the fully textile OECTs described in this report an extremely promising platform to realize wearable biosensors.

## Materials and Methods

### Materials

Clevios PH 1000 suspension (PEDOT:PSS) was purchased by Heraeus. Ascorbic Acid, Adrenaline, Dopamine, (3-Glycidyloxypropyl)trimethoxysilane (GOPS), Ethylene Glycol and Phosphate Buffer Saline (PBS) (x 10 concentrate) were purchased by Sigma Aldrich. PBS was used after the opportune dilution. All chemicals were reagent grade or higher. All solutions were prepared with distilled water. Woven cotton (250 μm thick) and lycra were bought in local market and used as received.

### Apparatus

Two SourceMeters 2400 SMU (Keithley) were simultaneously employed in order to carry out the electrical measurements by applying source-drain (V_d_) and source-gate (V_g_) potentials and measuring the respective currents (I_d_, I_g_).

The electrical conductivity of the e-textile was evaluated by measuring sheet resistance with a 4-probe custom setup, where four aligned and evenly spaced (spacing 2.7 mm) conductive tips were connected to a Keithley 2400 SourceMeter^41^. The measurements were repeated at three different positions for each sample and the sheet resistance was calculated by applying a geometrical correction factor of π/ln 2.

### OECT fabrication

Preparation of PEDOT:PSS ink for screen printing: 1% (w/v) of GOPS was added to a solution obtained by mixing two parts of Clevios PH 1000 and one part of ethylene glycol. About 40% (w/w) of the solvent was evaporated in order to obtain a liquid with the suitable viscosity for being used as an ink for screen printing.

Preparation of OECT printed on the fabric: the pristine textile was covered by using insulating tape as a mask. The PEDOT:PSS ink was applied on the edge of the textile and was moved onto the textile by the use of a fill blade. The tape was removed and the textile was dried in an oven at 60 °C for 15 min. A thin layer of PDMS was applied in order to partly cover the PEDOT:PSS track to keep dry the electrical contacts between the organic transistor channel material and the metallic electrodes of the electronic readout.

### Testing of the OECT performance

The OECT sensor was tested by two different experiments. Firstly the device performance was evaluated using ideal working condition, i.e. as the non-wearable OECT sensors are usually characterized. The OECT was fabricated with a geometry suitable (G1) for this kind of characterization (Fig. 2B). The OECT was dipped until the PDMS border reached the level of the PBS solution (25 mL) (Fig. 2D). Then, after the stabilization of I_d_, different amounts of analyte solutions were gradually added to electrolyte under magnetic stirring.

The second set of experiments was carried out in conditions that are closer to real use in a garment. The geometry (G2), reported in Fig. 2C, was employed in this case. 10 μL of PBS solution were added only on one side of the fabric in the area between the channel and the gate, and after the biasing of the source, drain and gate terminals and the stabilization of I_d_, different amounts of target compound were added in the same area (Fig. 2E).

## Additional Information

**How to cite this article**: Gualandi, I. *et al*. Textile Organic Electrochemical Transistors as a Platform for Wearable Biosensors. *Sci. Rep.* **6**, 33637; doi: 10.1038/srep33637 (2016).

## Supplementary Material

Supplementary Information

## Figures and Tables

**Figure 1:**
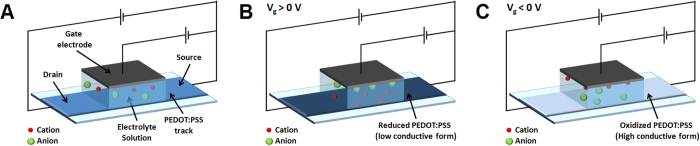
OECT working principle. Scheme of an OECT (**A**) operating in conditions of low (**B**) and high (**C**) conductivity of the channel.

**Table 1:** Electrical resistance of different devices reported in literature.

	Resistance	Reference
PEDOT screen printed	38.7 Ω/□ or 130 Ω cm^−1^	Our work
Carbon nanotube cotton yarn	500 Ω cm^−1^	15
Printed carbon electrode	20 Ω/□	24
Pedot:pss yarn/OECT	16.7–70 kΩ cm^−1^	35
Pedot:tos/Au nanoparticles yarn/OECT	25 kΩ cm^−1^	22
Pedot:pss yarn /OECT	400 Ω cm^−1^	30
Patterned PEDOT:PSS/electrocardiography electrode	230 Ω/□	20

**Table 2:** Textile OECT performance.

Geometry	Non-textile (on glass)		Textile		
	G1		G1		G2
	Linear range (M)	Sensitivity^a^ (LOD)^c^	Linear range (M)	Sensitivitya (LOD)^c^	Sensitivity^b^ (LOD)^d^
Ascorbic Acid	10^−6^–10^−3^	0.12 ± 0.01 (1.3 10^−8^)	10^−4^–10^−2^	0.37 ± 0.03 (1 10^−5^)	0.78 ± 0.05 (2.0)
Adrenaline	10^−5^–2 10^−4^	0.067 ± 0.007 (2 10^−6^)	3 10^−5^–5 10^−4^	0.75 ± 0.07 (1 10^−5^)	0.8 ± 0.1 (1.2)
Dopamine	10^−6^–10^−4^	0.10 ± 0.01 (5 10^−8^)	2 10^−6^–3 10^−5^	1.0 ± 0.2 (1 10^−6^)	1.1 ± 0.1 (0.4)

Parameters of calibration plots obtained in ideal conditions (geometry G1) and in real-life conditions (geometry G2) for a textile OECT and for an identical device fabricated on glass (ref. 31).

^a^Expressed as decade^−1^.

^b^Expressed as 10^5^ C mol^−1^.

^c^Expressed as M.

^d^Expressed as 10^−8^ mol.

**Table 3:** Adsorbed power of some wearable device reported in literature.

	Analyte	Trasduction	Adsorbed power	Ref.
Quantum Cascade Lasers	NO	Optical	1 mW	13
Adhesive RFID sensor bandage	Na^+^	Potentiometric	0 mW	40
Mouthguard biosensor	Uric Acid	Amperometric	0.7 mW^a^	17
Textile OECT/Ag Gate	Electrolyte	OECT sensor	0.08 mW^b^	36
Textile OECT/ Pt Gate	Adrenaline	OECT sensor	0.08 mW^b^	30
Fully-textile OECT G1	Redox compounds	OECT sensor	0.15 mW^b^	This work
Fully-textile OECT G2	Redox compounds	OECT sensor	0.15 mW^b^	This work

^a^Adsorbed power in standby mode (i. e. without data transfer).

^b^Electrical energy consumption due to the current that flows in the transistor channel.
