# Supplementary material for: Textile Organic Electrochemical Transistors as a Platform for Wearable Biosensors
Source: Sci Rep. 2016 Sep 26;6:33637. doi: 10.1038/srep33637 (PMC5035988; doi:10.1038/srep33637)
Supplement: Supplementary Information [file srep33637-s1.pdf]

Supplementary Materials

TEXTILE ORGANIC ELECTROCHEMICAL TRANSISTORS AS A PLATFORM FOR WEARABLE BIOSENSORS

I. Gualandi[1], M. Marzocchi[1], A. Achilli[2], D. Cavedale[1], A. Bonfiglio[2], B. Fraboni[1]

Figure S1. Picture of the transistor during bending

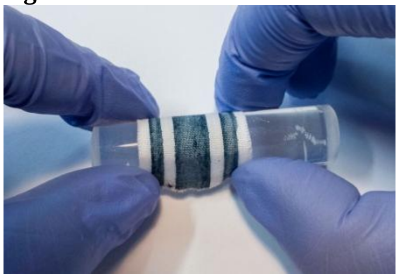

Figure S2. Resistance value of the transistor before, during and after bending. The error bars correspond to 3 standard deviation of average.

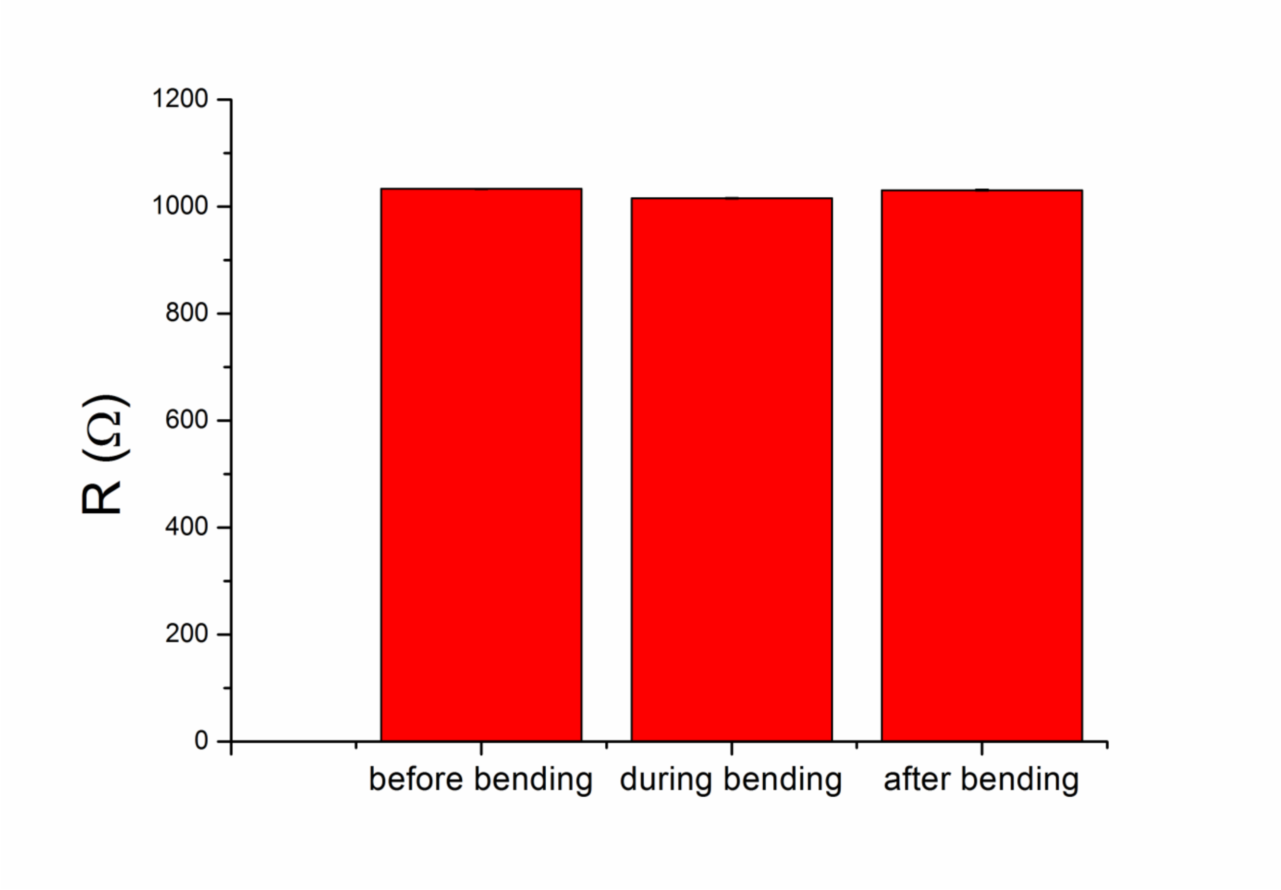

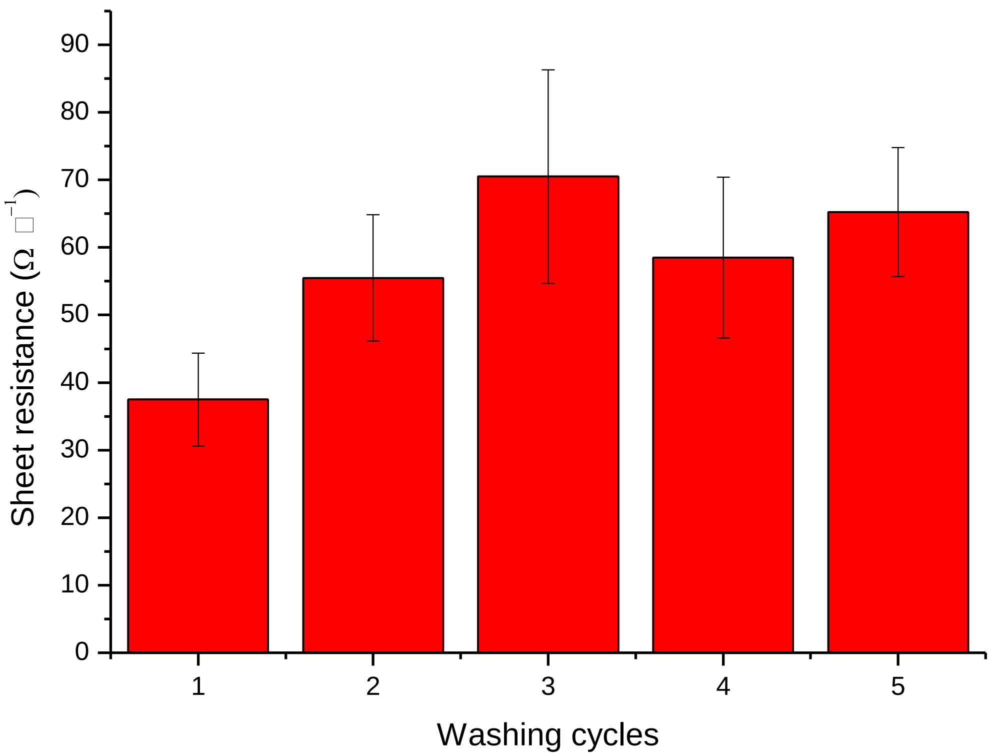

Fig. S3. **Resistance to washing.** Sheet resistance of the PEDOT:PSS track obtained by screen printing as a function of the number of washing cycles

Fig. S4 Characteristic curves recorded for a G1 transistor

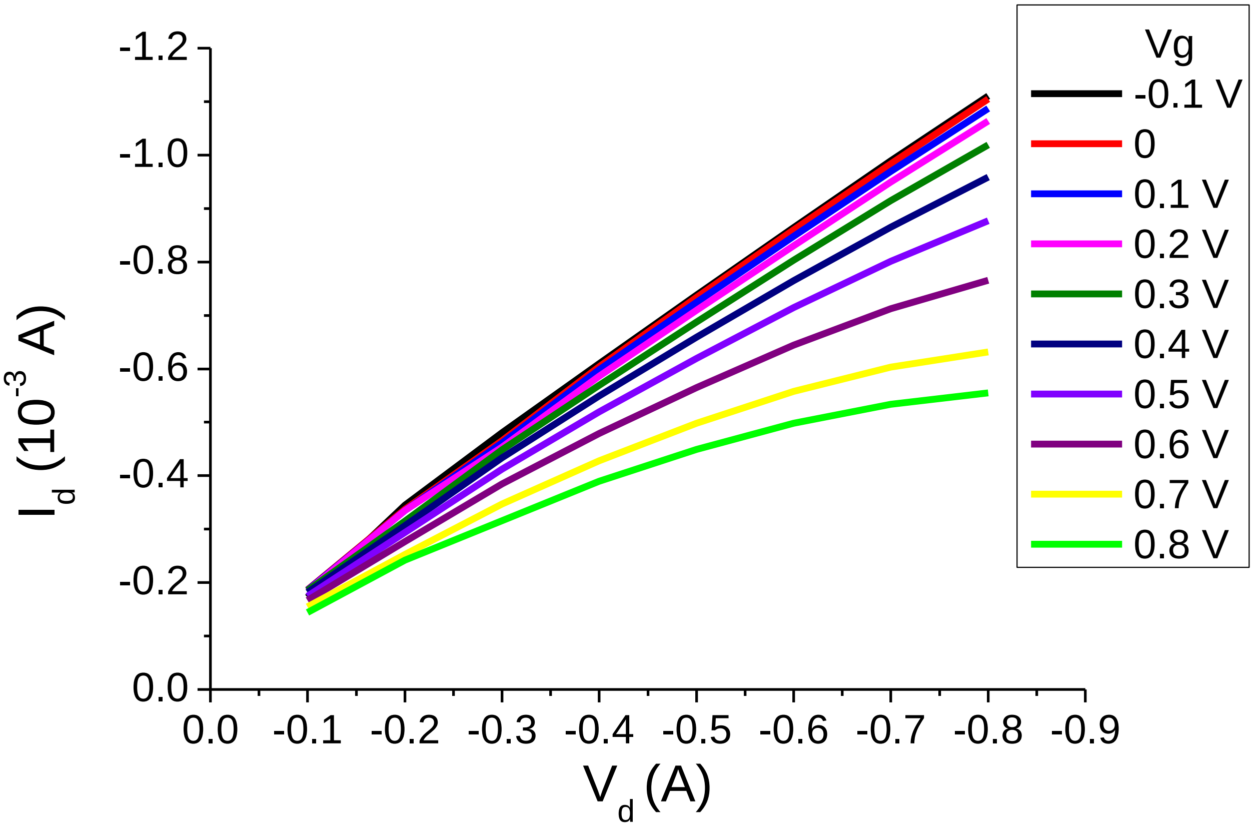

Figure S5. A) I_d in function of time for G1 OECT obtained while different amounts of ascorbic acid were added to the electrolytic solution. B) 1 – I/I_{max} versus Log C plot

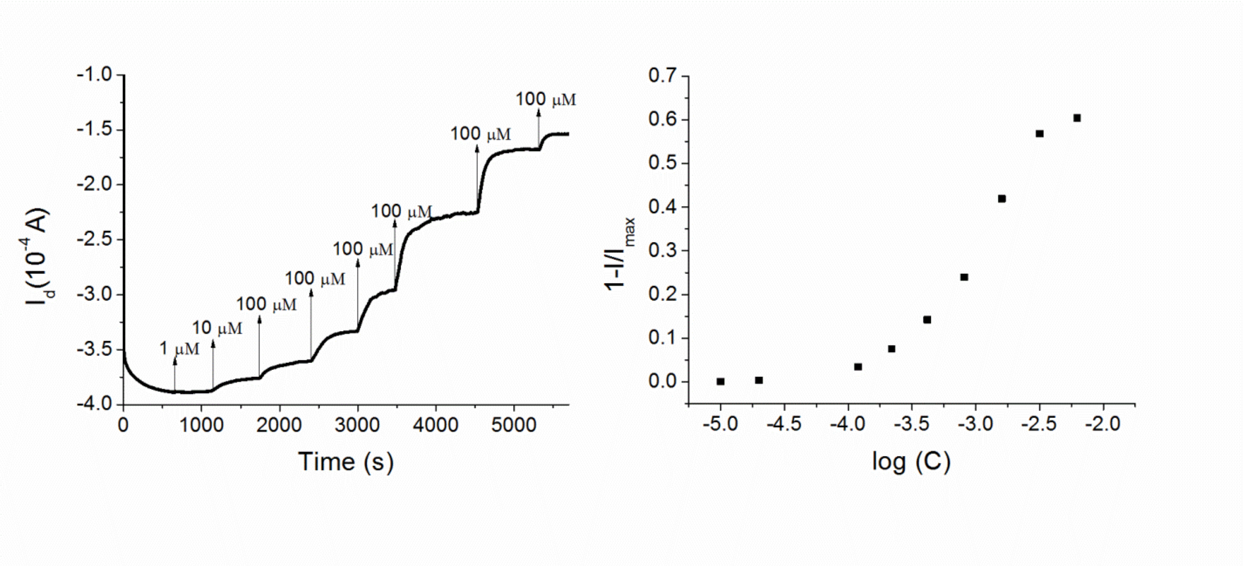

Figure S6. A) I_d in function of time for G1 OECT obtained while different amounts of dopamine were added to the electrolytic solution. B) 1 – I/I_{max} versus Log C plot

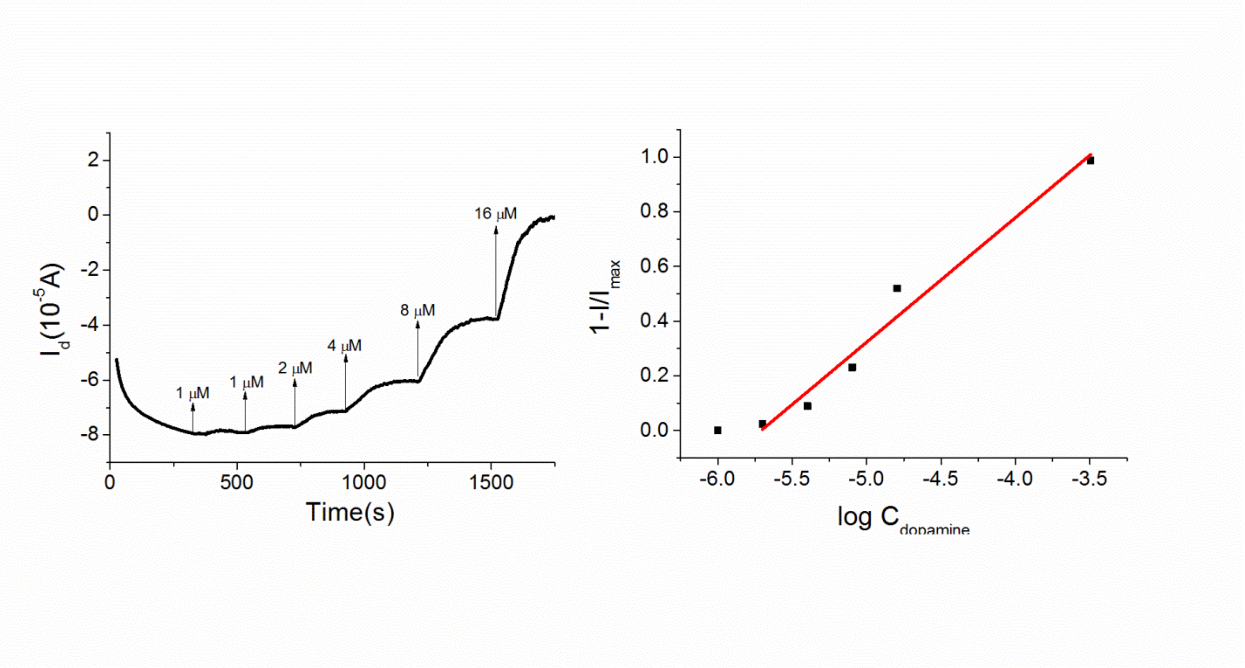

Fig SI7. Cyclic voltammetry recorded in PBS using as working electrode a stripe of PEDOT:PSS deposited onto glass slide. The red voltammogram was recorded in 0.1 mM adrenaline solution, while the black line is the voltammogram recorded in blank solution. The peak potential of adrenaline oxidation process is 0.090 V.

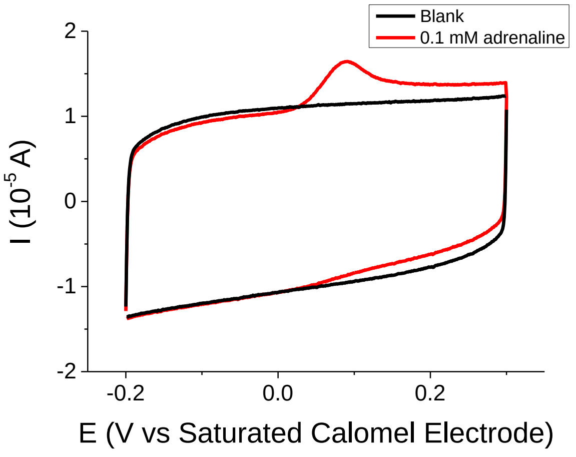

Figure S8. Characteristic curve of G2 OECT

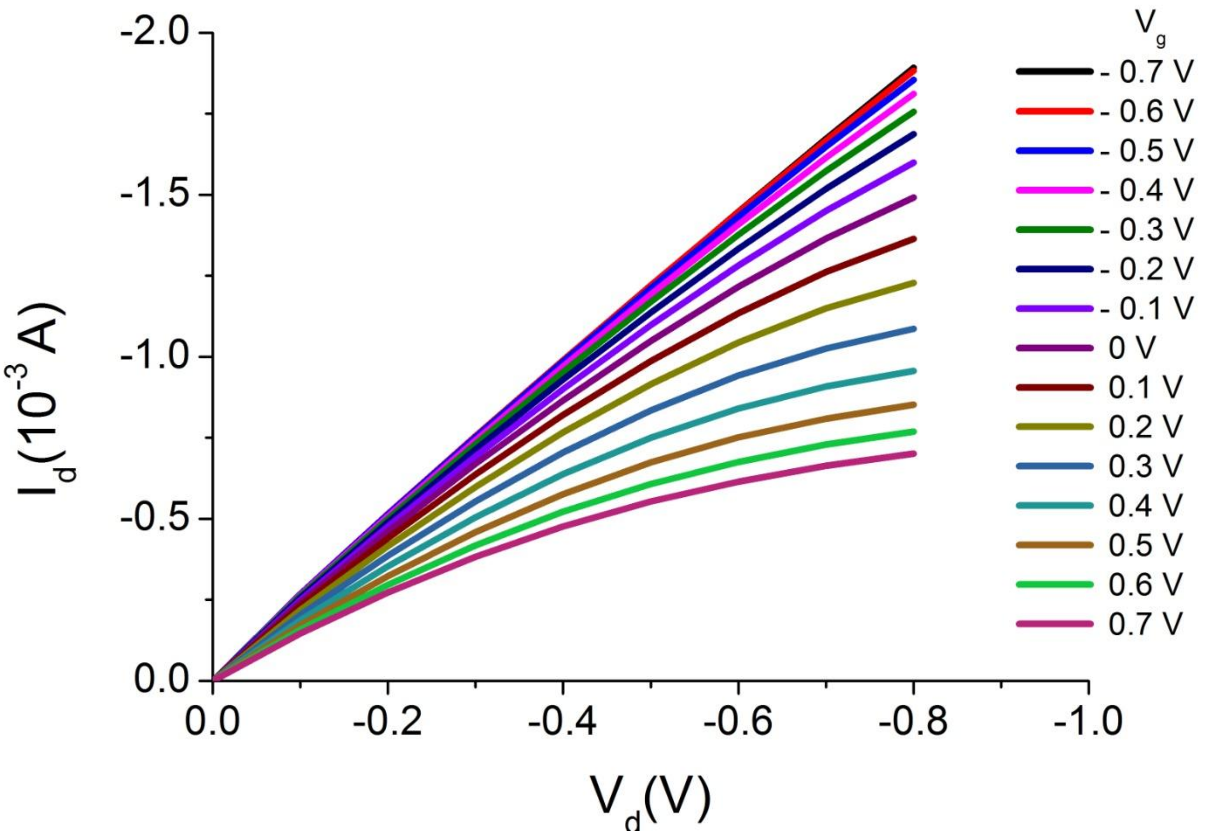

Figure S9. A) I_d in function of time for G2 OECT obtained while different amounts of ascorbic acid were added to the electrolytic solution. B) Charge versus concentration plot

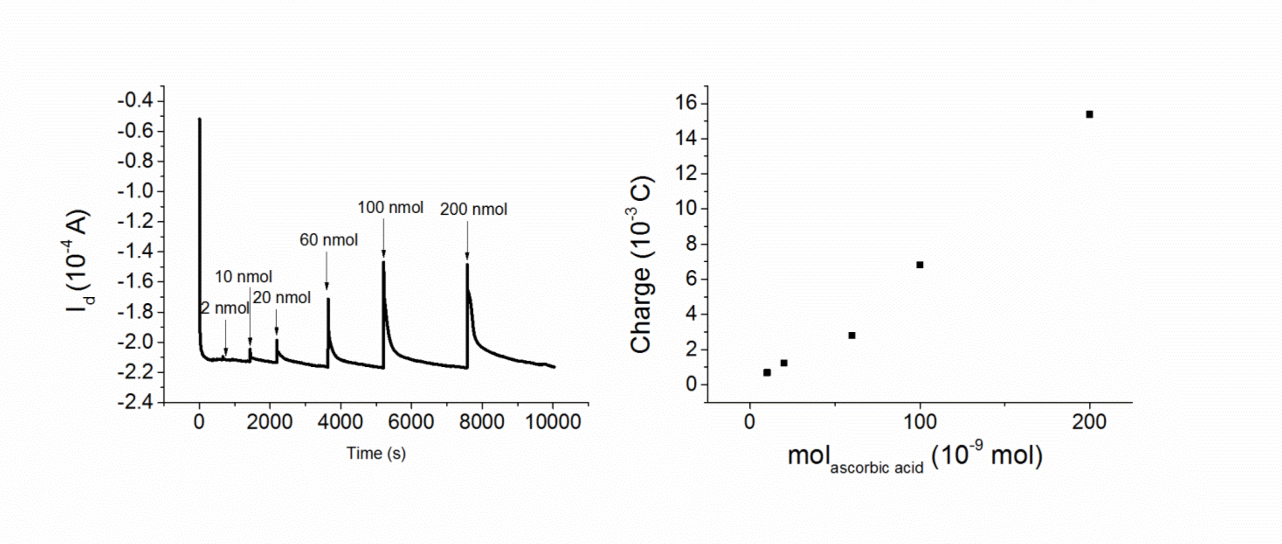

Figure S10. A) I_d in function of time for G2 OECT obtained while different amounts of adrenaline were added to the electrolytic solution. B) Charge versus Concentration plot

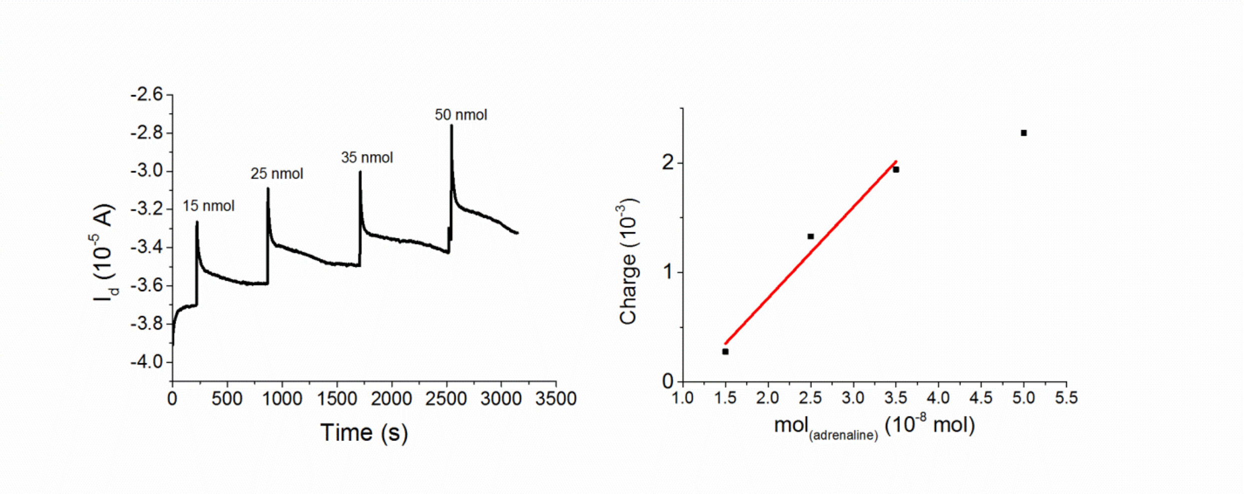

Table SI 1. Parameters of calibration plots obtained in artificial sweat

	Slope ($decade^{-1}$)	intercept	R^2
Ascorbic Acid	0.48 ± 0.02	1.48 ± 0.05	0.996
Adrenaline	0.28 ± 0.04	1.3 ± 0.2	0.94
Dopamine	0.40 ± 0.05	1.9 ± 0.2	0.95

Table SI 2. Experimental charge that flows at gate electrode after dopamine additions vs the theorical charge generated by dopamine oxidation.

**Experimental charge (mC)**	**Theoretical charge (mC)**
**0.56**	**0.96**
**1.4**	**1.9**
**2.0**	**2.9**
**2.8**	**3.8**
**2.5**	**4.8**
